# Supplementary material for: RN7SK small nuclear RNA controls bidirectional transcription of highly expressed gene pairs in skin
Source: Nat Commun. 2021 Oct 7;12:5864. doi: 10.1038/s41467-021-26083-4 (PMC8497571; doi:10.1038/s41467-021-26083-4)
Supplement: Supplementary file 1 — Supplementary Information [file 41467_2021_26083_MOESM1_ESM.pdf]

Supplementary Figure 1

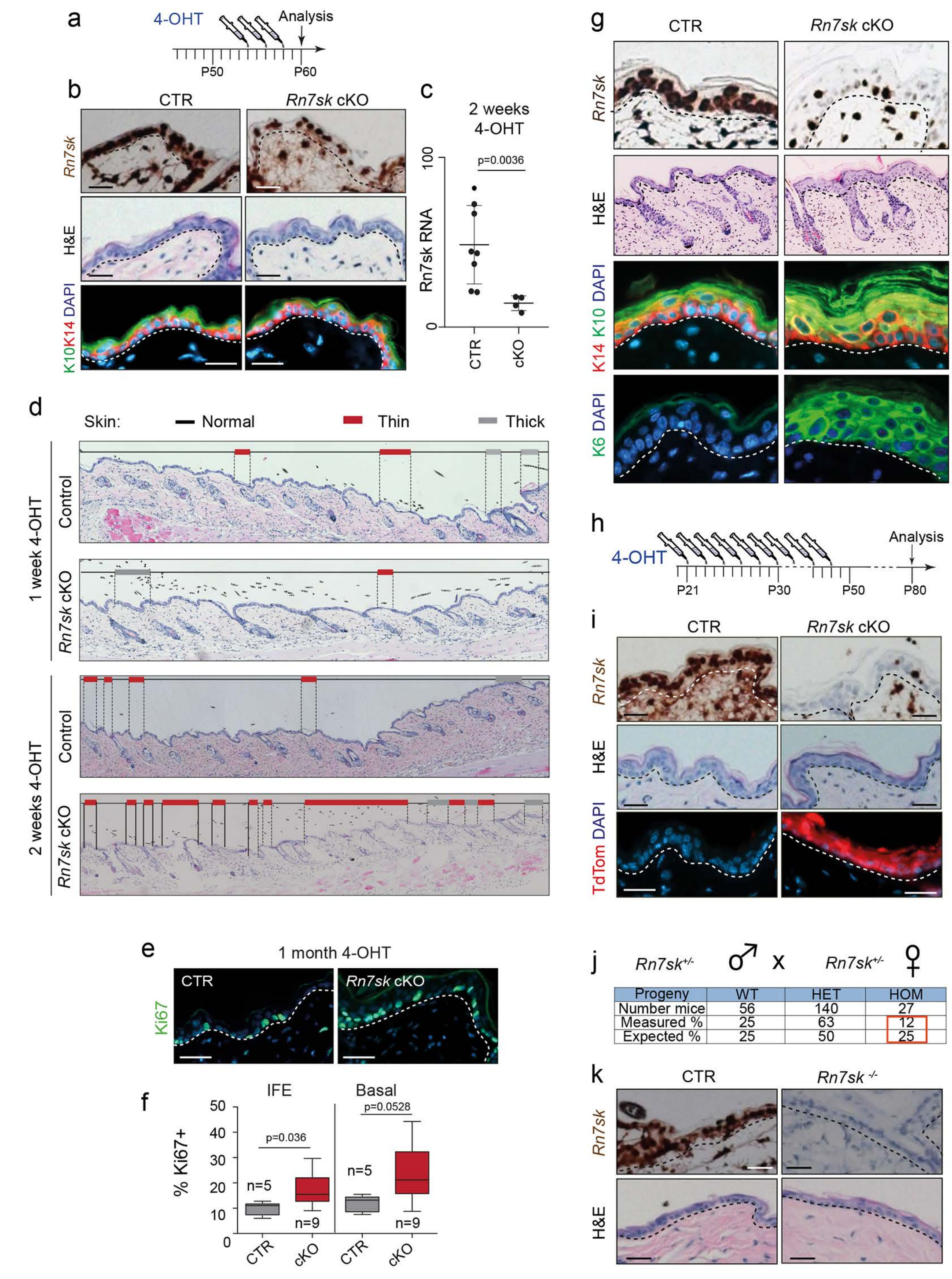

Progeny	WT	HET	HOM
Number mice	56	140	27
Measured %	25	63	12
Expected %	25	50	25

Figure S1. **Loss of cellularity induces wound-like response in the epidermis**. (**a**) Treatment regime of experiment shown in (b). (**b**) *Rn7sk* RNA *in situ* hybridisation (brown; top panel), haematoxylin and eosin staining (H&E, middle panel) and KRT10 (green) and KRT14 (red) immunofluorescence (bottom panel). (**c**) *Rn7sk* RNA levels in mouse skin after 2 weeks of 4-OHT exposure in control (CTR; n = 8 mice) and *Rn7sk* cKO (cKO; n = 4 mice) animals. (**d**) H&E staining from mouse skin treated with 4-OHT for 1 or 2 weeks, showing how skin thickness was quantified in Figure 1. Black stroke: normal; red bar: thin skin; grey bar: thick skin. (**e**, **f**) Ki67 (green) immunofluorescence (e) and quantification of Ki67-positive cells in IFE and basal layer (f) in control (grey; CTR) and *Rn7sk* ckO (red) mice after 1 month 4-OHT treatment (n = mice). (**g**) *Rn7sk* RNA *in situ* hybridisation (brown; top panels); H&E staining (2nd row panels); KRT10 (K10; green) and KRT14 (K14; red) (3rd row panels) and KRT6 (K6; green) (lower panels) immunofluorescence in mouse skin after 1 month 4-OHT treatment. (**h**) Treatment regime of experiment shown in (i). (**i**) *Rn7sk* RNA *in situ* hybridisation (brown; top panel), H&E staining (middle panel) and TdTomato (tdTom; red) immunofluorescence (bottom panel). Data generated from mouse line 1 are shown in (a-i). (**j**) Number and percent of mice per genotype obtained from crossing heterozygous *Rn7sk* +/- mice. (**k**) *Rn7sk* RNA *in situ* hybridisation (brown; top panel), H&E staining (bottom panel) of skin from surviving adult *Rn7sk* -/- and control mice. DAPI (blue): nuclear counterstain (b,e,g,i). Shown are presentative images from at least three different mice per genotype (b,e,g,i,k). Scale bars: 10 μm. Shown is mean ± SD. Unpaired two-tailed t test (f) with Welch's correction (c). Box plots in (f) show all points and mean with whiskers ending at minimum and maximum values. Exact p-values are indicated. Source data are provided as a Source Data file.

Supplementary Figure 2

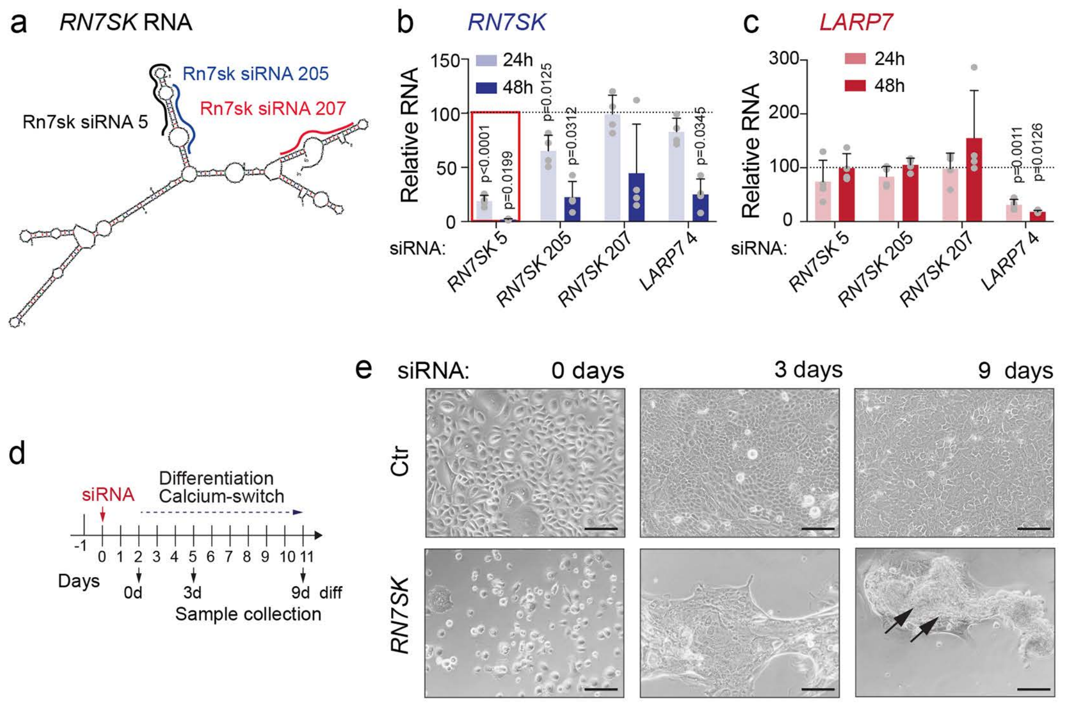

Figure S2. ***RN7SK*-depletion accelerates differentiation of human primary keratinocytes**. (**a**) Predicted *RN7SK* secondary structure determined using mfold (http://unafold.rna.albany.edu/?q=mfold). *RN7SK* siRNAs 5 (black), 205 (blue), 207 (red) targeted sequences are highlighted. (**b**, **c**) RNA levels of *RN7SK* (blue) and *LARP7* (red) in primary human keratinocytes after 24 (light blue or red) or 48 (dark blue or red) hours (h) of transfection with the indicated siRNAs. Data are normalised to *GAPDH* and expressed as relative to control siRNA (n = 4 transfections). (**d**) Treatment regime of human primary keratinocytes in days (d) shown in (e). (**e**) Bright field images of human keratinocytes transfected with *RN7SK* or control (Ctr) siRNAs during calcium-induced differentiation at the indicated time points. Arrows highlight enhanced stratification. Scale bars: 100 μm. Data are presented as mean values ± SD (b,c). Unpaired t test with Welch's correction (b,c). Exact p-values are indicated. Source data are provided as a Source Data file.

Supplementary Figure 3

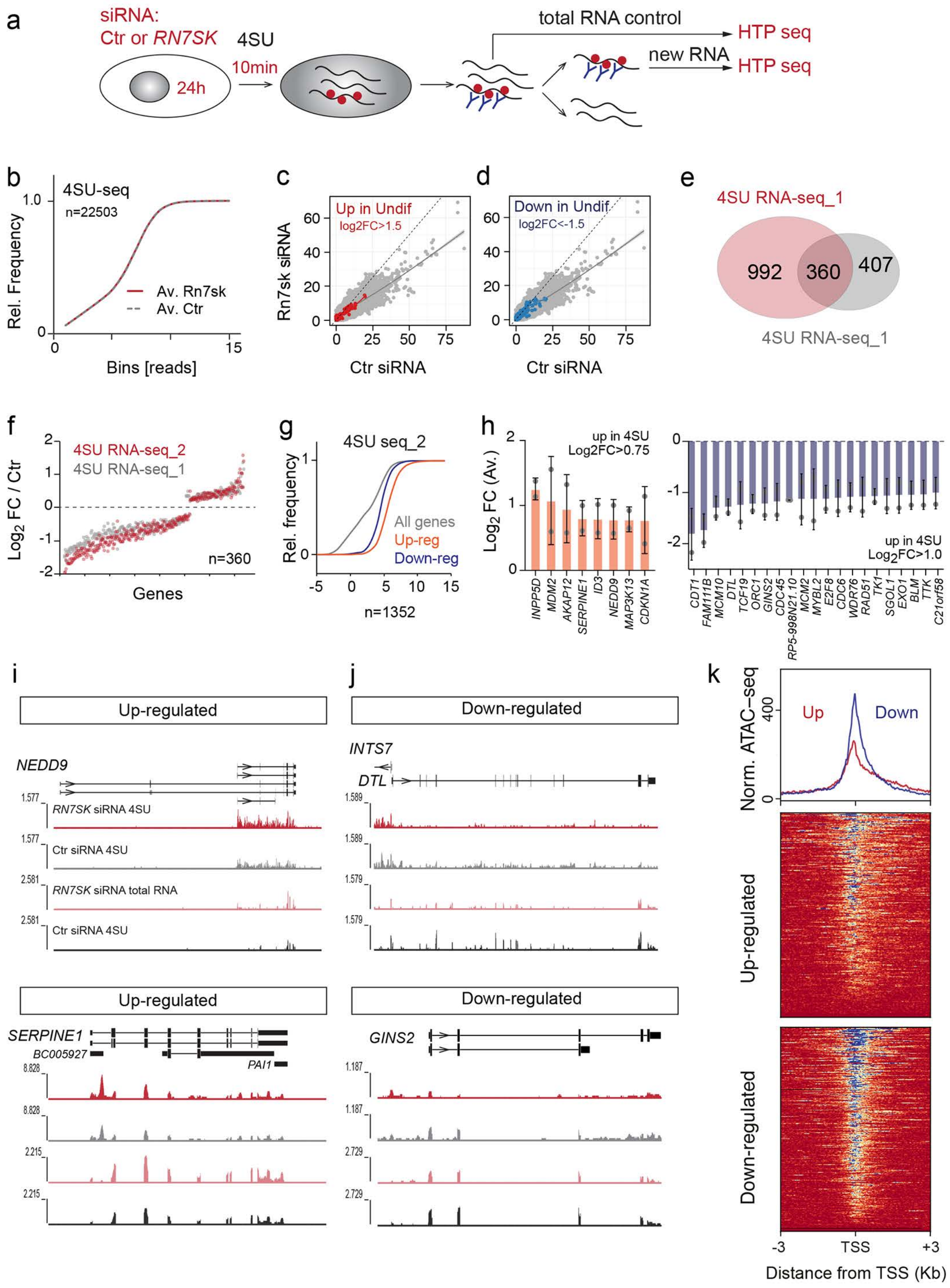

Figure S3. ***RN7SK* maintains expression levels of highly expressed genes**. (**a**) Schematic representation of metabolic RNA labelling experiments: primary human epidermal cells were transduced with control (Ctr) or *RN7SK* siRNAs for 24 hours. New RNA was labelled with 4SU for 10 minutes (min). High throughput sequencing (HTP seq) was performed on labelled and total unlabelled RNA. (**b**) Cumulative relative (rel.) frequency of average (Av.) sequence read counts (n = 4 sequencing reactions) of all genes in control (Ctr; grey) or *RN7SK*-depleted (red) cells. (**c, d**) Plot of RNA Pol II pausing index of up- (c) and down- (d) regulated genes in primary human keratinocytes transduced with Ctr or *RN7SK* siRNAs. Each dot represents one gene. (**e, f**) Overlap (e) and $\log_2$ fold-change (FC) (f) of significantly different new transcripts (padj<0.05) in two independent 4SU seq experiments. n = transcripts. (**g**) Cumulative rel. frequency of sequence read counts of all genes (grey) or up- (orange) or down- (blue) regulated new transcripts (4SU RNA seq_2). (**h**) Significantly different (padj<0.05) top up- (left panel; orange) and down- (right panel; blue) regulated new transcripts in two independent 4SU RNA sequencing experiments. Shown is the average $\log_2$ FC. (**i, j**) UCSC genome browser shots of 4SU and total RNA sequencing reads of the indicated up- (i) and down- (j) regulated genes. (**k**) Density plot (upper panel) showing ATAC seq normalized reads at transcription TSSs of up- (red) or down-regulated (blue) genes in 4SU RNA seq dataset at 48h. Heat maps (lower panels) showing ATAC seq signal at TSS of up- (top) or down-regulated (bottom) genes in 4SU RNA seq (padj < 0.05, $\text{Log}_2\text{Fc} > 0.3$).

Supplementary Figure 4

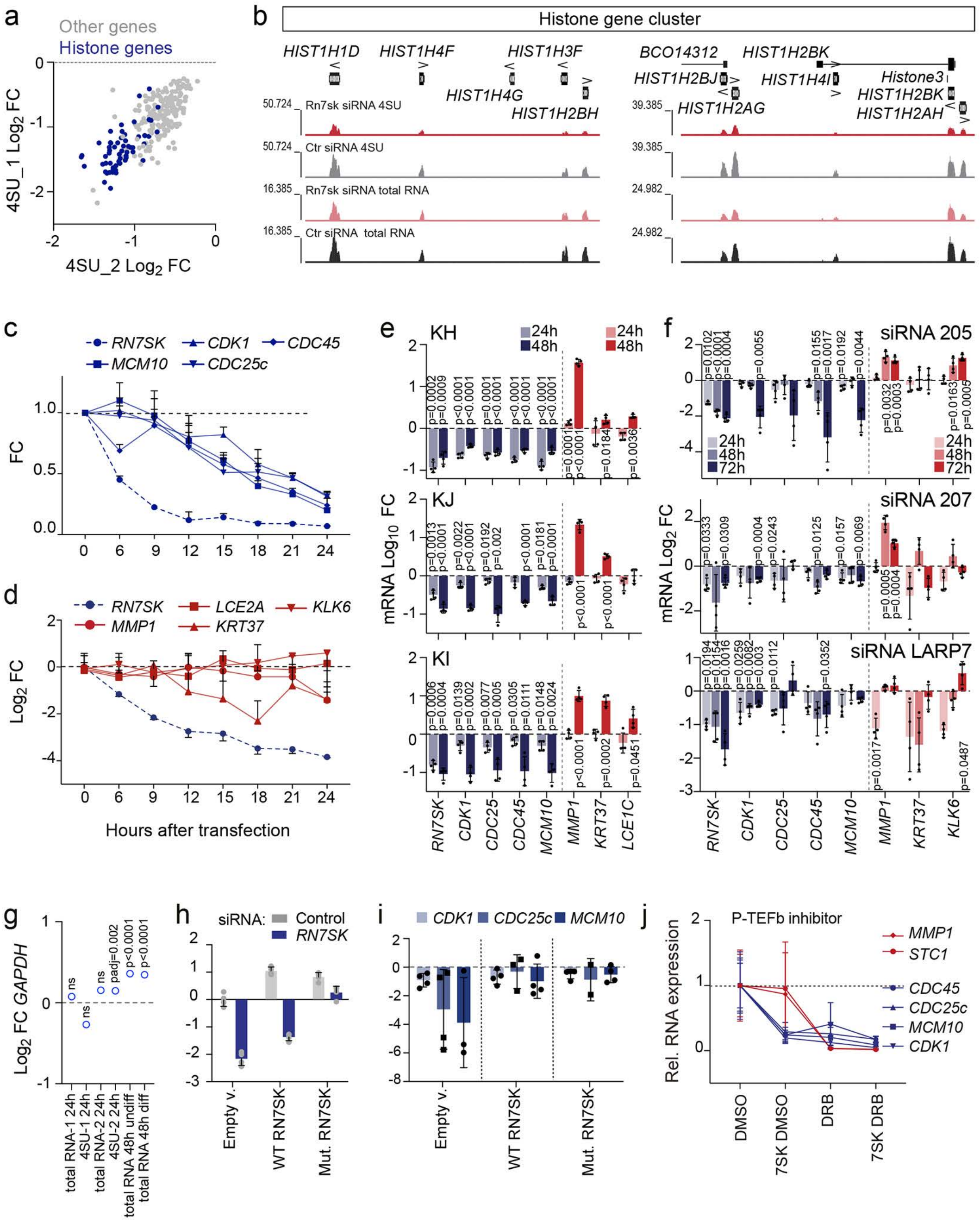

Figure S4. ***RN7SK* directly represses expression of cell cycle regulators**. (**a**) Significantly down-regulated newly transcribed histone gene (blue) in two independent 4SU RNA-seq datasets. Grey: Other genes. (**b**) UCSC genome browser shot showing example of histone gene cluster repressed in *RN7SK*-depleted cells. (**c, d**) RNA level fold-change (FC) of four down-regulated (blue) cell cycle regulators (c) and four up-regulated (red) genes (d) in primary human keratinocytes in a time course after knock-down of *RN7SK* (dotted line). (**e, f**) Log_{10} FC of RNA of the indicated genes down- (blue) or up- (red) regulated in three different primary human keratinocytes lines (KH, KJ, KI) (e) or using three different siRNAs (205, 207, *LARP7*) (f) 24 (light colour), 48 (darker colour) or 78 (darkest colour) hour (h) after control or indicated siRNA transfections. (**g**) Log_2 FC of Gapdh reads in the indicated RNA sequencing datasets. Ns: not significant. (**h, i**) *RN7SK* (h) or cell cycle regulators (i) RNA levels in human primary keratinocytes infected with constructs expressing wild-type or mutated *RN7SK* 24 h after control (grey) or *RN7SK* (blue) siRNA transfections. RNA levels in (i) are normalized to control siRNA transfected cells. (n = 4 transfections). (**j**) Relative (Rel.) RNA levels for the indicated up- (red) or down- blue) regulated RNAs in human keratinocytes transfected with control or *RN7SK* siRNAs for 24 hours and treated with DRB for the last 6 hours. RT-qPCR data are normalised to *GAPDH* and expressed as mean ± SD relative to the respective controls (n = 4 transfections) (c-f,h,i) or mean values ± SEM (j). Unpaired two-tailed t test with Welch's correction (e,f). Wald test (g). Exact p-values are indicated. Source data are provided as a Source Data file.

Supplementary Figure 5

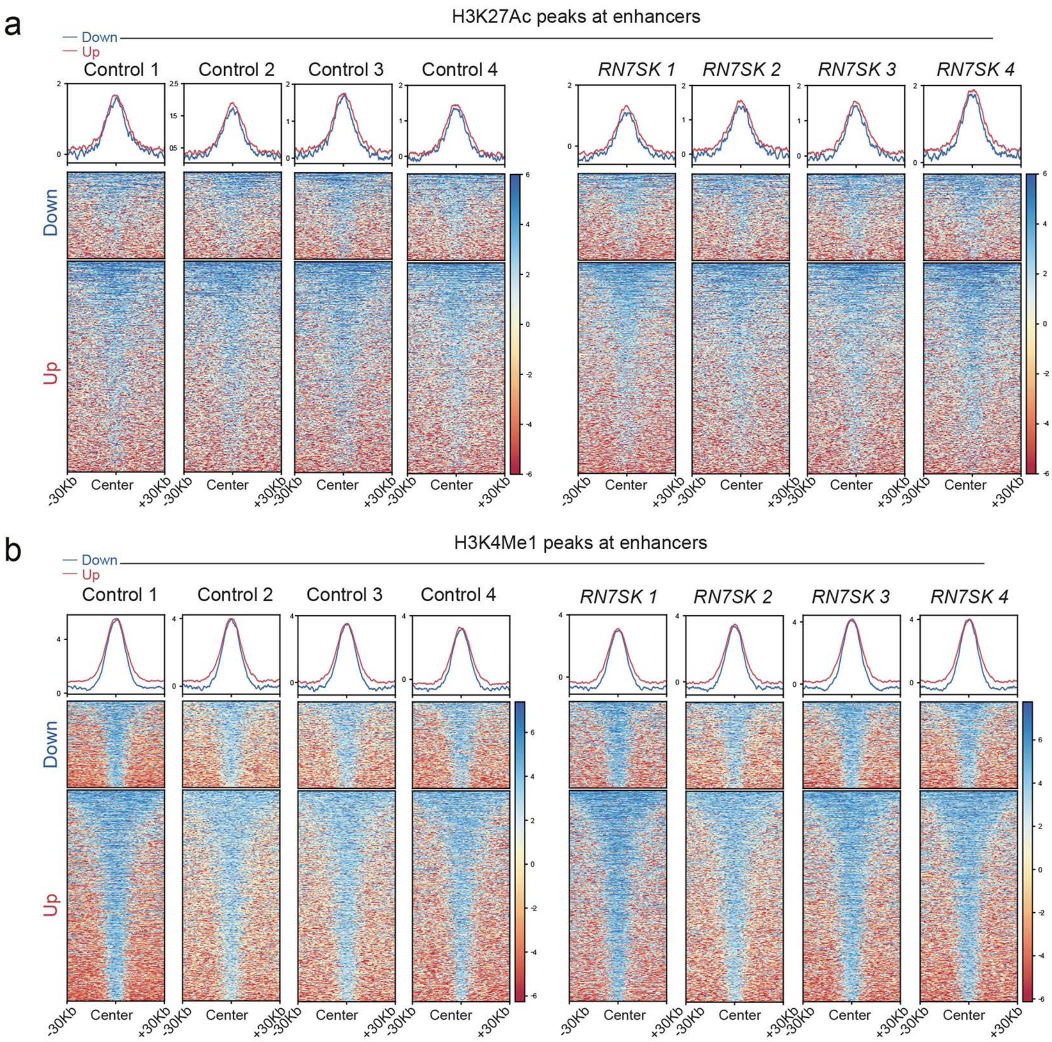

Figure S5. ***RN7SK*-depletion does not alter chromatin in promoters and putative enhancers**. (**a, b**) All replicate heatmaps showing H3K4Me1 (a) and H3K27Ac (b) putative enhancers (H3K4me1-positive and H3K27ac-positive peaks) close to the transcriptional start site (TSS) (< 30 Kb) of up- (red) and down- (blue) regulated genes.

Supplementary Figure 6

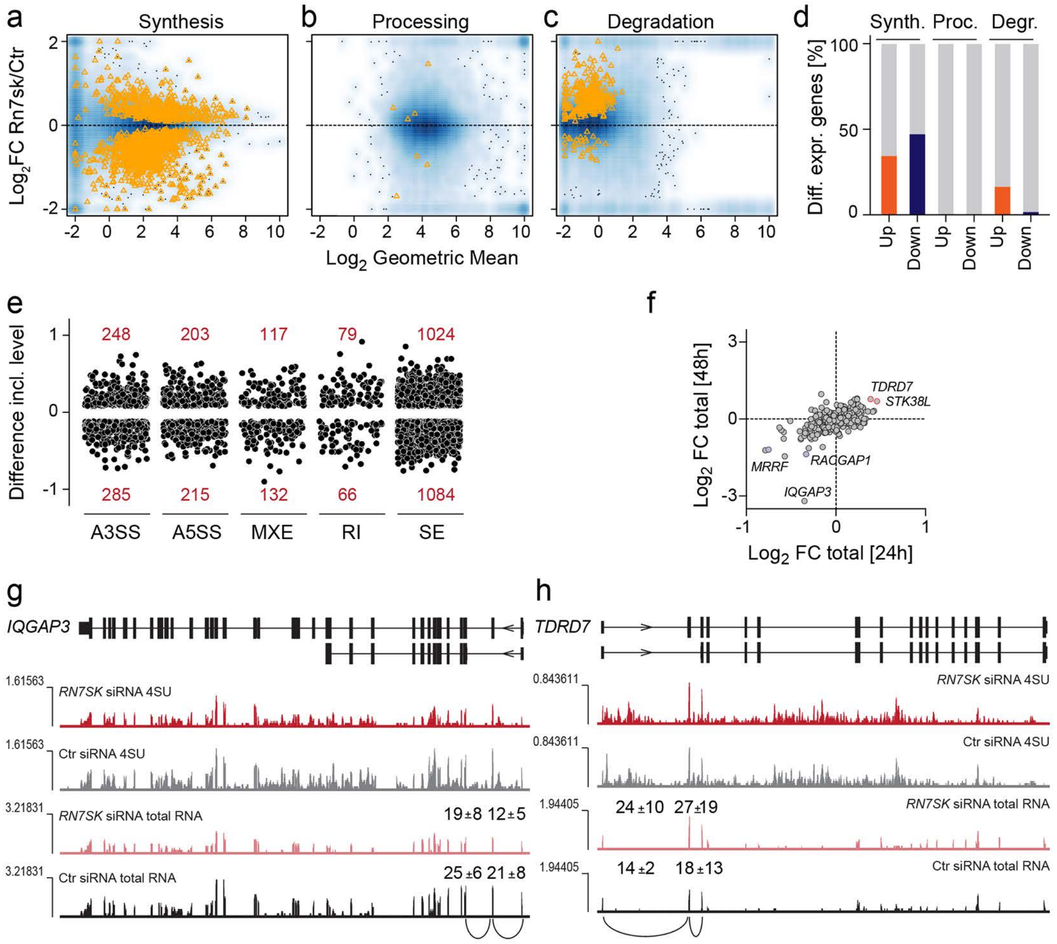

Figure S6. **Effect of *RN7SK*-depletion on RNA synthesis, splicing and degradation**. (**a-c**) Density scatter plots (darker colours for higher density) of $\log_2$ fold-changes (FC) in the absence of *RN7SK* versus time points (0 and 24 hours and 10 minutes RNA labelling time). Shown are changes in RNA levels caused by differences in RNA synthesis (a), processing (b) and mature mRNA degradation (c). Yellow triangle: one gene. Only genes with > = 20 counts per million are shown. (**d**) Quantification of (a-c). (**e**) Splicing differences in total RNA, 24 hours after *RN7SK*-depletion (splicing FDR>0.05; inclusion difference >0.1 or <-0.1). A3SS: alternative 3' splice site; A5SS: alternative 5' splice site; MXE: mutual exclusive exon; RI: intron retention; SE: exon skipping. (**f**) Correlation of $\log_2$ fold change (FC) RNA levels commonly found to be differentially spliced in two independent total RNA-seq datasets at 24 or 48 hours after *RN7SK* depletion. (**g, h**) Examples of alternatively spliced transcripts resulting in down- (g) or up- (h) regulation of the RNA shown as UCSC genome browser shots. Source data are provided as a Source Data file.

Supplementary Figure 7

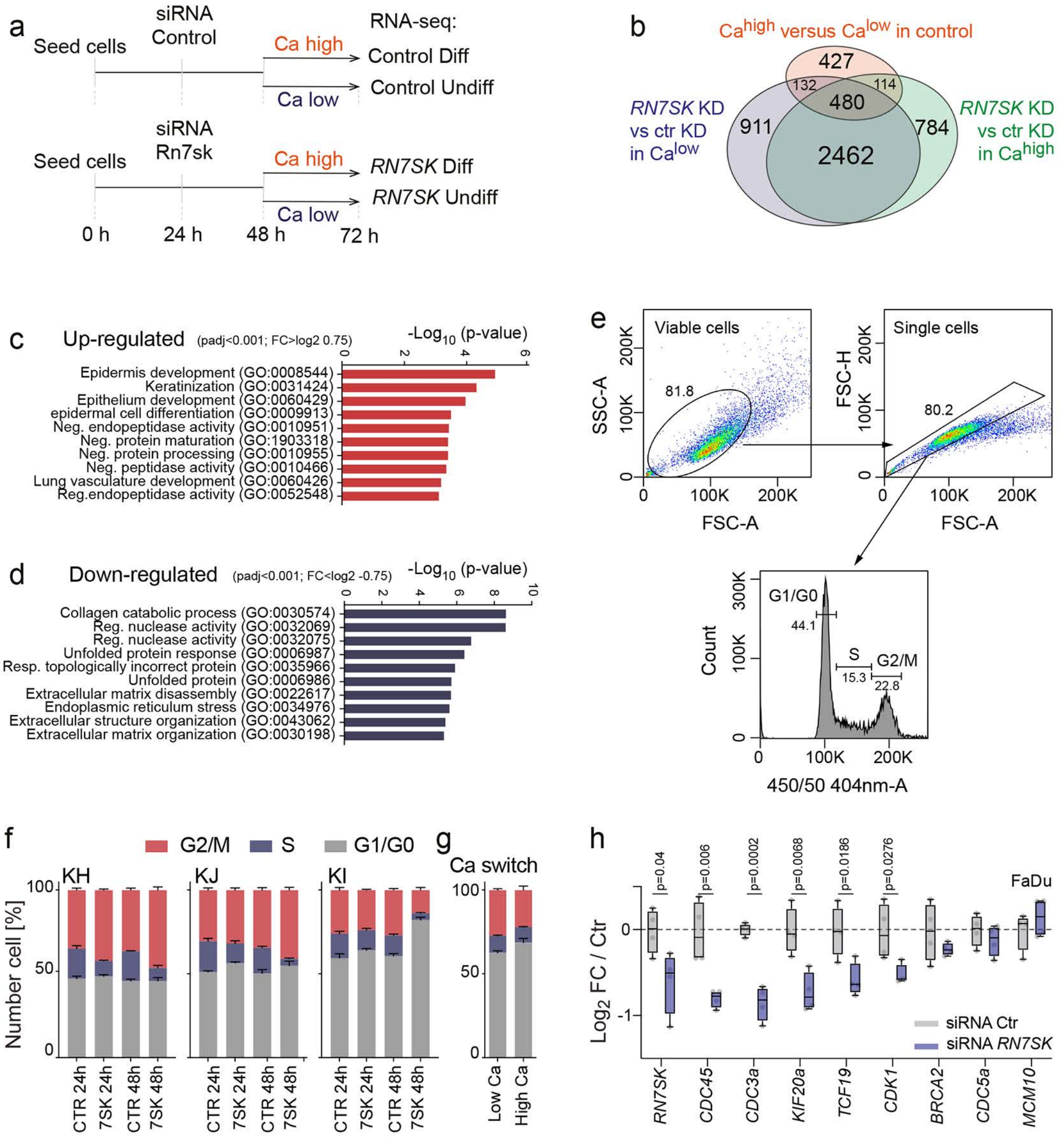

Figure S7. Differentiation of *RN7SK*-depleted keratinocytes is distinct from calcium-induced differentiation. (**a**) Experimental set-up and treatment regime of primary human epidermal cells for RNA sequencing experiments. (**b**) Venn diagram showing significantly changed genes (padj < 0.001) in the indicated datasets compared to control cells. KD: knock-down. (**c, d**) Gene ontology analysis of up- (c) or down-regulated (d) genes (padj<0.001; log_2FC> or < 0.75) in response to calcium differentiation (calcium switch) of human keratinocytes. (**e**) Gating strategy to determine the percentage of cells in cell cycle phases. (**f, g**) Quantification of cell cycle profile of different lines of human primary keratinocytes 24 or 48 hours (h) after siRNA transfection (f) or grown in calcium low or high conditions (n = 5 transfections) (g). (**h**) RT-qPCR for the indicated RNAs in *RN7SK*-depleted FaDu cells (blue). Data are normalised to 18s rRNA and expressed as mean relative to empty vector control (siRNA Ctr; grey) (n = 3 or 4 transfections). Data are presented as mean values ± SD (f,g). Box plots show all points and mean with whiskers ending at minimum and maximum values (i). Unpaired two-tailed t-test. Exact p-values are indicated. Source data are provided as a Source Data file.

Table S1. Primer sequences

Primer name	Sequence	Usage
oIMR0042	CTAGGCCACAGAATTGAAAGATCT	Genotyping KRT14 CRE ERT
oIMR0043	GTAGGTGGAAATTCTAGCATCATCC	Genotyping KRT14 CRE ERT
oIMR3797	ATACCGGAGATCATGCAAGC	Genotyping KRT14 CRE ERT
oIMR3798	AGGTGGACCTGATCATGGAG	Genotyping KRT14 CRE ERT
Tom WT fwd	AAG GGA GCT GCA GTG GAG TA	Genotyping Rosa:TdTomato
Tom WT rev	CCG AAA ATC TGT GGG AAG TC	Genotyping Rosa:TdTomato
Tom mut rev	GGC ATT AAA GCA GCG TAT CC	Genotyping Rosa:TdTomato
Tom mut fwd	CTG TTC CTG TAC GGC ATG G	Genotyping Rosa:TdTomato
7SK 3' ARM S	GGTCTACAGAGAAAGTCCC	Genotyping Rn7SK
7SK Targeting seq 14319 AS	TGAAGGTTCCAAGCAGTCG	Genotyping Rn7SK
D5 AS	GATGGCGCAACGCAATTAA	Genotyping Rn7SK
h7SK_P20_L	attgatcgccagggttgat	Q-PCR human Rn7sk
h7SK_P20_R	ctctatcggggatggtcgt	Q-PCR human Rn7sk
hGAPDH 5'	gagtccactggcgtcttcac	Q-PCR human GAPDH
hGAPDH 3'	ttcacacccatgacgaacat	Q-PCR human GAPDH
m7SK P109 L	tccattgtaggagaacgtaggg	Q-PCR mouse Rn7sk
m7SK P109 R	agcgcctcatttggatgt	Q-PCR mouse Rn7sk
mGAPDH 5'	tccactcatggcaaattcaa	Q-PCR mouse GAPDH
mGAPDH 3'	tttgatgttagtggggtctcg	Q-PCR mouse GAPDH
Cdc45 TSS s1	AAAAGGCCAGGCGAAGAC	MNAse protection assay
Cdc45 TSS s2	AATGCCCTTTCGTGATTT	MNAse protection assay
Cdc45 TSS s3	GGCCCATTGTCCTTACGA	MNAse protection assay
Cdc45 TSS s4	CGACTACACTACCCACAATGC	MNAse protection assay
Cdc45 TSS s5	TCGGGCGGAACTAAGCTA	MNAse protection assay
Cdc45 TSS s6	GCCCCATTGGGTATGTGA	MNAse protection assay
Cdc45 TSS s7	TGGGAGGCAGTAGGCTTC	MNAse protection assay
Cdc45 TSS s8	GCGTCGCTTTTCTGGGTA	MNAse protection assay
Cdc45 TSS s9	GAGGTGACGCTTCTTTGG	MNAse protection assay
Cdc45 TSS s10	TGAATGGCAGAGCGCTAA	MNAse protection assay
Cdc45 TSS s11	CTGGACCAATCGGAGGAG	MNAse protection assay
Cdc45 TSS s12	ATTTGGCGGGAGTCTTGA	MNAse protection assay
Cdc45 TSS s13	TACGAGGTGGTCCAGAGC	MNAse protection assay
Cdc45 TSS s14	GGTGGGGAAGGGATGAGG	MNAse protection assay
Cdc45 TSS s15	GTTCAGCCGGTCTGCTCT	MNAse protection assay
Cdc45 TSS s16	TGTGTGCGTGCAAGATCC	MNAse protection assay
Cdc45 TSS s17	CGGGCAGAGTGTCAGGAT	MNAse protection assay
Cdc45 TSS as1	AAATCACGAAAGGGCATT	MNAse protection assay
Cdc45 TSS as2	TGGGCCCTACTAAATTCGTC	MNAse protection assay
Cdc45 TSS as3	GAGTCCTCGATGGCTGAAG	MNAse protection assay
Cdc45 TSS as4	CTTAGTTCCGCCCGAGTC	MNAse protection assay
Cdc45 TSS as5	GGCAGCATTTACGTTTGC	MNAse protection assay
Cdc45 TSS as6	AGCCTACTGCCTCCCACT	MNAse protection assay
Cdc45 TSS as7	GTTGCCCCCTTCAACTCT	MNAse protection assay
Cdc45 TSS as8	CCCAAAGAAGCGTCACCT	MNAse protection assay
Cdc45 TSS as9	ATTGGAGGCGGCCATTAG	MNAse protection assay
Cdc45 TSS as10	CTCCTCCGATTGGTCCAG	MNAse protection assay
Cdc45 TSS as11	CGCGCTGAGGTACCAAGA	MNAse protection assay
Cdc45 TSS as12	CGGAAATCGGACACGAAC	MNAse protection assay
Cdc45 TSS as13	GCTCCCCCTCATCCCTTC	MNAse protection assay
Cdc45 TSS as14	CCTCTATCGGGGAAGAGC	MNAse protection assay
Cdc45 TSS as15	AAGGATCTTGCACGCACA	MNAse protection assay
Cdc45 TSS as16	ACGCAGCACCCTCACCTC	MNAse protection assay
Cdc45 TSS as17	GCAACCTCTCCTCCGAGT	MNAse protection assay
Mcm10 TSS s1	TCGGGAAATGGTGGTTTC	MNAse protection assay
Mcm10 TSS s2	TGGTGAAGGGGAAACTCA	MNAse protection assay
Mcm10 TSS s3	CACTTCCCCAAGGTCAGA	MNAse protection assay
Mcm10 TSS s4	GGACCGGATCTGTTTGGT	MNAse protection assay
Mcm10 TSS s5	GCGTCAGGAGTTGAGGTG	MNAse protection assay
Mcm10 TSS s6	CCGCAGGGAAAATACTGG	MNAse protection assay
Mcm10 TSS s7	GGGCGCCAGACACTCTAT	MNAse protection assay
Mcm10 TSS s8	GGCGTCCAATGACGTAAA	MNAse protection assay
Mcm10 TSS s9	CTGGCGTAGCCAGTCAGT	MNAse protection assay
Mcm10 TSS s10	TTGCTGGCCGTCTACTAA	MNAse protection assay
Mcm10 TSS s11	AGGAATCGGGTTCTTCCTTT	MNAse protection assay
Mcm10 TSS s12	GCCTGGGCTTTCTTTTATTC	MNAse protection assay
Mcm10 TSS s13	TTCATTCATTGGTTTCTCGT	MNAse protection assay
Mcm10 TSS as1	CTGAGTTTCCCCTTCACCA	MNAse protection assay
Mcm10 TSS as2	CAAACAGATCCGGTCCACTT	MNAse protection assay
Mcm10 TSS as3	GTCAGCCTTATGTCCATTCG	MNAse protection assay
Mcm10 TSS as4	GCTCTGGGACCGACTGTT	MNAse protection assay

Mcm10 TSS as5	CTGGTGCCGGGAAGTTTA	MNAse protection assay
Mcm10 TSS as6	GGGACCTTCCTGTCGGTA	MNAse protection assay
Mcm10 TSS as7	GGACGCCCTCTTTGTTGG	MNAse protection assay
Mcm10 TSS as8	AGCACTGACTGGCTACGC	MNAse protection assay
Mcm10 TSS as9	ACCCGCCAAAATTCCAAC	MNAse protection assay
Mcm10 TSS as10	AGGCACTTGGCAAGCACT	MNAse protection assay
Mcm10 TSS as11	CCAATGAATGAATGAATGAATG	MNAse protection assay
Mcm10 TSS as12	ACCGTCCGCATTCAATAATC	MNAse protection assay
Mcm10 TSS as13	GATCCGAGCCATGCACAC	MNAse protection assay
Cdk1 TSS s1	ACATTTTTGAGGCGGTCT	MNAse protection assay
Cdk1 TSS s2	CCATTTCCTTCTTAAGGTCAC	MNAse protection assay
Cdk1 TSS s3	TCACGCATTTAGAAAAACATAA	MNAse protection assay
Cdk1 TSS s4	GGAATCTCGATGTAAACACAA	MNAse protection assay
Cdk1 TSS s5	TTGAACTGTGCCAATGCT	MNAse protection assay
Cdk1 TSS s6	GAAGAACGGAGCGAACAG	MNAse protection assay
Cdk1 TSS s7	TTGCGCTCGCACTCAGTT	MNAse protection assay
Cdk1 TSS s8	TTCTTTCGCGCTCTAGCC	MNAse protection assay
Cdk1 TSS s9	GCGTAGCTGGGCTCTGAT	MNAse protection assay
Cdk1 TSS s10	CCTTTAGCGCGGTGAGTT	MNAse protection assay
Cdk1 TSS s11	GAAACTGCTCGCACTTGG	MNAse protection assay
Cdk1 TSS s12	GCGACGCGGTTGTTGTAG	MNAse protection assay
Cdk1 TSS s13	GGGTCAGGGTCGTGTCTA	MNAse protection assay
Cdk1 TSS s14	CACTGGGCTGGCTTTAGA	MNAse protection assay
Cdk1 TSS s15	GTTCGGGGCTGCAGTCAT	MNAse protection assay
Cdk1 TSS s16	AGTCGAGGTCGGCTGTTC	MNAse protection assay
Cdk1 TSS as1	GGCTCCAAGGAGCACATTT	MNAse protection assay
Cdk1 TSS as2	TGTTTTTCTAAATGCGTGATT	MNAse protection assay
Cdk1 TSS as3	TGTGTTTACATCGAGATTCCA	MNAse protection assay
Cdk1 TSS as4	CAGCATTGGCACAGTTCAA	MNAse protection assay
Cdk1 TSS as5	GCGGAGCAGGAAGCTACT	MNAse protection assay
Cdk1 TSS as6	CCAGTCGGGAGAGTGTCG	MNAse protection assay
Cdk1 TSS as7	GGTGGCTAGAGCGCGAAA	MNAse protection assay
Cdk1 TSS as8	GCCCGTAGACTTTCAAAGCA	MNAse protection assay
Cdk1 TSS as9	AACTCACCGCGCTAAAGG	MNAse protection assay
Cdk1 TSS as10	TCGCTCTCCGCTCAATTT	MNAse protection assay
Cdk1 TSS as11	GCGGCAGCTACAACAACC	MNAse protection assay
Cdk1 TSS as12	GCCGTCCCCTAGACACGA	MNAse protection assay
Cdk1 TSS as13	CCAGTCCGGCCTTCTTAG	MNAse protection assay
Cdk1 TSS as14	ATGACTGCAGCCCCGAAC	MNAse protection assay
Cdk1 TSS as15	CAATCAAGACCCGAAAGC	MNAse protection assay
Cdk1 TSS as16	GGAGCGGATTTTGCTTCA	MNAse protection assay
Cdc25c TSS s1	AGTCTCTGAGATGCTGCACAC	MNAse protection assay
Cdc25c TSS s2	CATAAACACACACACCCGTCT	MNAse protection assay
Cdc25c TSS s3	GGCCAGCAAAACTTAGAAGAA	MNAse protection assay
Cdc25c TSS s4	ACACCCTCCACCCAAAATAA	MNAse protection assay
Cdc25c TSS s5	GTTTAAATCTCCCGGGGTTC	MNAse protection assay
Cdc25c TSS s6	AGGGAGAGCCAATGATGC	MNAse protection assay
Cdc25c TSS s7	GGCCAAACACTATCCTGCTC	MNAse protection assay
Cdc25c TSS s8	GTGATCTGCGAGCCCAAC	MNAse protection assay
Cdc25c TSS s9	AGCGGGGATAGGTTACTGG	MNAse protection assay
Cdc25c TSS s10	TAACTTTGGCCTTCTGCTCA	MNAse protection assay
Cdc25c TSS s11	GAAGGCAGAGCTGCAATCTA	MNAse protection assay
Cdc25c TSS s12	CCTGTGTCCGATCCCTATCT	MNAse protection assay
Cdc25c TSS s13	GTAAGTCCGCAGCCGTCTC	MNAse protection assay
Cdc25c TSS s14	CCCCACAGACAGAGGTTGG	MNAse protection assay
Cdc25c TSS as1	ACGGGTGTGTGTGTTTATGC	MNAse protection assay
Cdc25c TSS as2	CTAAGTTTTGCTGGCCTTCC	MNAse protection assay
Cdc25c TSS as3	TTATTTTGGGTGGAGGGTGT	MNAse protection assay
Cdc25c TSS as4	GATTTAAACCGCGCCTTACT	MNAse protection assay
Cdc25c TSS as5	TCTCCCTCTCACCAATCTCC	MNAse protection assay
Cdc25c TSS as6	CCATAGCCAGAGCAGGATAG	MNAse protection assay
Cdc25c TSS as7	GTTGGGCTCGCAGATCAC	MNAse protection assay
Cdc25c TSS as8	CTAAGCTGCGTCAGCCAAT	MNAse protection assay
Cdc25c TSS as9	AGTTACGGCCTCTGAGCAAG	MNAse protection assay
Cdc25c TSS as10	ATTGCAGCTCTGCCTTCC	MNAse protection assay
Cdc25c TSS as11	AGATAGGGATCGGACACAGG	MNAse protection assay
Cdc25c TSS as12	CTGCGGACTTACCTCAAGC	MNAse protection assay
Cdc25c TSS as13	TCCCAACCTCTGTCTGTGG	MNAse protection assay
Cdc25c TSS as14	CACAGCTCGAGACTCCTGAC	MNAse protection assay

Table S2. Antibodies used in this study

Antibody	Company	Catalogue number	Species	Diluition	Usage	Research ResourceIdentifier (RRID)
Keratin 14	Covance	PRB-155P-100	Rabbit	1:1000	IF	AB_292096
Keratin 10	Santa Cruz	sc23877	Mouse	1:200	IF	AB_2134668
Keratin 6	Abcam	Ab24646	Rabbit	1:200	IF	AB_448211
Keratin 6	Thermo Fisher	MS-766-P0	Mouse	1:100	IF	AB_141613
Ki67	Vector Labs	VP-RM04	Rabbit	1:200	IF	AB_2336545
RNA pol II N-20	Santa Cruz	sc-899	Rabbit	10ug per C	ChIP	AB_632359
H3K4Me1	Abcam	Ab8895	Rabbit	5ug per Ch	ChIP	AB_306847
H3K27Ac	Abcam	Ab4729	Rabbit	10ug per C	ChIP	AB_2118291
RNA Pol II P Ser 2	MBL	MABI0602	Mouse	1:2000	Westen Blot	AB_2747403
RNA Pol II P Ser 5	MBL	MABI0603	Mouse	1:2000	Westen Blot	AB_2728736
RNA Pol II total CTD	MBL	MABI0601	Mouse	1:2000	Westen Blot	AB_2728735
Vinculin	Abcam	ab129002	Rabbit	1:10000	Westen Blot	AB_11144129
Goat anti-Rabbit IgG (H+L) Highly Cross-Adsorbed Secondary Antibody, Alexa Fluor Plus 488	Thermo Fisher Scientific	A32731	Goat	1:500	IF	AB_2633280)
Goat anti-Mouse IgG (H+L) Highly Cross-Adsorbed Secondary Antibody, Alexa Fluor Plus 555	Thermo Fisher Scientific	A32727	Goat	1:500	IF	AB_2633276
Goat anti-Mouse IgG (H+L) Secondary Antibody, HRP	Thermo Fisher Scientific	31430	Goat	1:10000	Westen Blot	AB_228307
Goat anti-Rabbit IgG (H+L) Secondary Antibody, HRP	Thermo Fisher Scientific	65-6120	Goat	1:10000	Westen Blot	AB_2533967

Table S3. SiRNA constructs and summary of time points.

siRNA	ENSEMBL ID	siRNA name	Company
Control	N/A	AllStars neg ctr	Qiagen
Rn7SK	ENSG00000283293	7SK siRNA 5	Qiagen
Rn7SK	ENSG00000283293	7SK siRNA 205	Thermo Fisher
Rn7SK	ENSG00000283293	7SK siRNA 207	Thermo Fisher
Larp7	ENSG00000174720	Larp7 siRNA 4	Qiagen

siRNA	Experiment	Time point (after transfection)
Rn7sk	RNA Pol II ChIPseq	18 hours
	H3K4Me1 ChIPseq	18 hours
	H3K27Ac ChIPseq	18 hours
	Total RNAseq low or high calcium	48 hours
	4SU/Total RNAseq_1	24 hours
	4SU/Total RNAseq_2	24 hours

Table S4. Taqman probes used in this study

Taqman probe	Target gene name	Species
Human GAPDH CTR	Gapdh	Human
Hs00277883_m1	Larp7	Human
Hs00902520_m1	Involucrin	Human
Hs01070316_m1	Tgm1	Human
Hs01041013_m1	Itga6	Human
Hs00899658_m1	Mmp1	Human
Hs00938777_m1	Cdk1	Human
Hs00160519_m1	Klk6	Human
Hs00174970_m1	STC1	Human
Hs00156411_m1	CDC25C	Human
Hs00960349_m1	MCM10	Human
Hs01596213_g1	KRT37	Human
Hs00820278_sH	LCE2A	Human
Hs00907337_m1	CDC45	Human
Mm00486872_m1	Cdc25c	Mouse
Mm00772472_m1	Cdk1	Mouse
Mm00486893_m1	Cdc45	Mouse
Mm01171453_m1	CcnB2	Mouse
Mm00712529_m1	Mcm10	Mouse
