# Supplementary material for: RN7SK small nuclear RNA controls bidirectional transcription of highly expressed gene pairs in skin
Source: Nat Commun. 2021 Oct 7;12:5864. doi: 10.1038/s41467-021-26083-4 (PMC8497571; doi:10.1038/s41467-021-26083-4)
Supplement: Supplementary file 3 — Reporting Summary [file 41467_2021_26083_MOESM3_ESM.pdf]

Corresponding author(s): Susanne Bornelöv and Michaela Frye

Last updated by author(s): Aug 13, 2021

Reporting Summary

Nature Portfolio wishes to improve the reproducibility of the work that we publish. This form provides structure for consistency and transparency in reporting. For further information on Nature Portfolio policies, see our Editorial Policies and the Editorial Policy Checklist.

Statistics

For all statistical analyses, confirm that the following items are present in the figure legend, table legend, main text, or Methods section.

n/a	Confirmed	
☐	☒	The exact sample size (n) for each experimental group/condition, given as a discrete number and unit of measurement
☐	☒	A statement on whether measurements were taken from distinct samples or whether the same sample was measured repeatedly
☐	☒	The statistical test(s) used AND whether they are one- or two-sided<br>*Only common tests should be described solely by name; describe more complex techniques in the Methods section.*
☒	☐	A description of all covariates tested
☐	☒	A description of any assumptions or corrections, such as tests of normality and adjustment for multiple comparisons
☐	☒	A full description of the statistical parameters including central tendency (e.g. means) or other basic estimates (e.g. regression coefficient) AND variation (e.g. standard deviation) or associated estimates of uncertainty (e.g. confidence intervals)
☐	☒	For null hypothesis testing, the test statistic (e.g. F, t, r) with confidence intervals, effect sizes, degrees of freedom and P value noted<br>*Give P values as exact values whenever suitable.*
☒	☐	For Bayesian analysis, information on the choice of priors and Markov chain Monte Carlo settings
☐	☒	For hierarchical and complex designs, identification of the appropriate level for tests and full reporting of outcomes
☐	☒	Estimates of effect sizes (e.g. Cohen's d, Pearson's r), indicating how they were calculated

Our web collection on statistics for biologists contains articles on many of the points above.

Software and code

Policy information about availability of computer code

Data collection	All brightfield and immunofluorescence images were acquired using an Axio Imager 2 Upright Research Microscope (Zeiss) with ORCA-Flash 4.0 digital camera (Hamamatsu) or AxioCam MRc colour digital camera (Zeiss). Flow cytometry analysis was performed with the LSRFortessa Flow Cytometer (BD Biosciences). RT-qPCR data acquisition was conducted using the StepOnePlus Real-Time PCR System (Applied Biosystems). Data were collated in Microsoft Excel (v16.16.27).
Data analysis	All images were initially processed using the ZEN Microscope and Imaging Software, and stored as TIFF files using Adobe Photoshop (v21.2.9). ImageJ 1.52q was used for image quantifications and Prism (GraphPad Softwares inc; v9.2.0) was used for data analysis. Flow cytometry data was analysed using the FlowJo software (FlowJo LLC; v10.6.2). Sequencing data analyses were performed using RStudio. All images and figures were arranged using Adobe Illustrator (v24.3). The bioinformatics analysis was done using a combination of publicly available software: Trim Galore!, TopHat2, featureCounts, DESeq2, Bowtie, MACS2, rMATs, bedops, and deepTools. Custom scripts developed in the study are available on GitHub (https://github.com/susbo/Bandiera-et-al-2021-scripts).

For manuscripts utilizing custom algorithms or software that are central to the research but not yet described in published literature, software must be made available to editors and reviewers. We strongly encourage code deposition in a community repository (e.g. GitHub). See the Nature Portfolio guidelines for submitting code & software for further information.

Data

Policy information about availability of data

All manuscripts must include a data availability statement. This statement should provide the following information, where applicable:

- Accession codes, unique identifiers, or web links for publicly available datasets
- A description of any restrictions on data availability
- For clinical datasets or third party data, please ensure that the statement adheres to our policy

All next-generation sequencing data generated as part of this study are available through GEO (GSE101217). ATAC-seq data from human keratinocytes are available through GEO (GSE67382; sample GSM1645708 and GSM1645709). CAGE-seq data from human keratinocytes are available through the FANTOM5 Table Extraction Tool (sample FF:11349-117G8, FF:11421-118F8, and FF:11272-116H3; http://fantom.gsc.riken.jp/5/tet). All original data are provided in the source data file.

Field-specific reporting

Please select the one below that is the best fit for your research. If you are not sure, read the appropriate sections before making your selection.

☒ Life sciences ☐ Behavioural & social sciences ☐ Ecological, evolutionary & environmental sciences

For a reference copy of the document with all sections, see nature.com/documents/nr-reporting-summary-flat.pdf

Life sciences study design

All studies must disclose on these points even when the disclosure is negative.

Sample size	Animal experiments: For the phenotypical and histological analyses our study used a minimum of 5 animals per experimental group. The number of mice was estimated by considering the use of the animals, like breeding and crossing and by considering smallest number of mice to achieve the highest benefit in results that is statistically necessary. In our experience using the specific conditional knock-out strain, 5 mice per genotype is sufficient (e.g. Driskell et al. 2012 EMBO J). Less than 5 mice may have been used when a time course was performed. For quantification experiments, each animal will only be measured once. For immunofluorescence analyses, the quantification will be automated to avoid bias by for example using ImageJ, which will measure up to 100 technical replicates. An average of technical replicates represents one biological replicate.<br><br>Experiments using cells: For targeted and genome-wide gene expression analyses of cultured cells, our study used a minimum of 4 replicates (one replicate = one transfection). This sample size was estimated using previous NGS datasets (e.g. Blanco et al. 2016 Nature; Selmi et al. 2021 NAR). The sample sizes considered that some sequencing methods were not standard (e.g. 4SU) and quality of the libraries varies. Less than 4 replicates may have been used when a time course was analyzed. RT-qPCRs were additionally performed in at least 3 technical replicates. An average of technical replicates represented one biological replicate.
Data exclusions	No data were excluded from the analysis.
Replication	An experiment involving animals was at least replicated twice. Functional assays involving cells in culture was at least replicated twice (CFE; DED-assay; Western Blot etc). RNA-seq and 4SU-seq experiments were repeated twice. ChIP-seq was performed once and not repeated because we observed no differences. In addition, Rn7sk-deletion phenotype was confirmed by two different mouse lines crossed to two different conditional deleter strains (K14CreER and Sox2Cre). Knock-down in cells was confirmed using three different siRNAs targeting Rn7sk and three independent keratinocytes cell lines.
Randomization	Mice were randomly allocated to each time point after the genotype was determined. For cell culture experiments, cells were equally distributed into multi-plate wells and the treatment condition was randomly applied. No additional controls for covariates was performed as the mice were age- and gender-matched and cells from the same passages were used for the experiments.
Blinding	Blinding was not possible because the genotype of the mice was known.

Reporting for specific materials, systems and methods

We require information from authors about some types of materials, experimental systems and methods used in many studies. Here, indicate whether each material, system or method listed is relevant to your study. If you are not sure if a list item applies to your research, read the appropriate section before selecting a response.

Materials & experimental systems

n/a	Involved in the study
☐	☒ Antibodies
☐	☒ Eukaryotic cell lines
☒	☐ Palaeontology and archaeology
☐	☒ Animals and other organisms
☒	☐ Human research participants
☒	☐ Clinical data
☐	☐ Dual use research of concern

Methods

n/a	Involved in the study
☐	☒ ChIP-seq
☐	☒ Flow cytometry
☒	☐ MRI-based neuroimaging

Antibodies

Antibodies used

All antibodies are also listed in Supplementary Table 2:
Keratin 14 Covance PRB-155P-100 Rabbit 1:1000
Keratin 10 Santa Cruz sc23877 Mouse 1:200
Keratin 6 Abcam Ab24646 Rabbit 1:200
Keratin 6 Thermo Fisher MS-766-P0 Mouse 1:100
Ki67 Vector Labs VP-RM04 Rabbit 1:200
RNA pol II N-20 Santa Cruz sc-899 Rabbit 10ug per ChIP
H3K4Me1 Abcam Ab8895 Rabbit 5ug per ChIP
H3K27Ac Abcam Ab4729 Rabbit 10ug per ChIP
RNA Pol II P Ser 2 MBL MABI0602 Mouse 1:2000
RNA Pol II P Ser 5 MBL MABI0603 Mouse 1:2000
RNA Pol II total CTD MBL MABI0601 Mouse 1:2000
Vinculin Abcam ab129002 Rabbit 1:10000
Goat anti-Rabbit IgG (H+L) Highly Cross-Adsorbed Secondary Antibody, Alexa Fluor Plus 488 Thermo Fisher Scientific A32731 Goat 1:500
Goat anti-Mouse IgG (H+L) Highly Cross-Adsorbed Secondary Antibody, Alexa Fluor Plus 555 Thermo Fisher Scientific A32727 Goat 1:500
Goat anti-Mouse IgG (H+L) Secondary Antibody, HRP Thermo Fisher Scientific 31430 Goat 1:10000
Goat anti-Rabbit IgG (H+L) Secondary Antibody, HRP Thermo Fisher Scientific 65-6120 Goat 1:10000

Validation

All antibodies have been used and validated in prior studies. In this study, all antibodies were validated for their expression and correct localization to the specific epidermal layers on mouse and human skin sections. All antibodies were used according to the manufacturer, who has validated the antibodies before. Secondary antibody only controls were used to confirm specificity.

Eukaryotic cell lines

Policy information about cell lines

Cell line source(s)

Neonatal primary human keratinocytes (ScienCell; #2100 - distributed by distributed by Cellworks UK). FaDu cells were obtained from ATCC (HTB-43)

Authentication

Validation was performed through expression of epidermis-specific keratin markers.

Mycoplasma contamination

All cell lines tested negative for mycoplasma.

Commonly misidentified lines
(See ICLAC register)

No commonly misidentified cell lines were used in this study.

Animals and other organisms

Policy information about studies involving animals; ARRIVE guidelines recommended for reporting animal research

Laboratory animals

All transgenic lines were bred on a mixed background (F1 of B6SJL x CBA). The inducible Cre lines (KRT14-cre/ERT)20Efu/J; JAX 005107 and B6N.Cg-Edil3Tg(Sox2-cre)1Amc/J; JAX 014094) and the reporter line (Gt(ROSA)26Sortm9(CAG-tdTomato)Hze; JAX 007909) were obtained from The Jackson Laboratory. The generation of the Rn7sk cKO transgenic lines is described in this publication. Both male and female mice were used. Experiments were performed with age- and gender-matched animals. All mice were group housed under specific pathogen-free conditions in individually ventilated cages always with companion mice, and cages were placed under a 12 hours light-dark cycle. Food and water were provided ad libitum. Room temperature was maintained at 22°C ± 1°C with 30–70% humidity. None of the mice were involved in any previous procedures before the study.

Wild animals

The study did not involve wild animals.

Field-collected samples

The study did not involve samples collected from the field.

Ethics oversight

This research has been regulated under the Animals (Scientific Procedures) Act 1986

Ethics oversight	Amendment Regulations 2012 following ethical review and approval by the University of Cambridge Animal Welfare and Ethical Review Body (AWERB) under the terms of the UK Home Office license PPL80/2619 and PPLP36B3A804.

Note that full information on the approval of the study protocol must also be provided in the manuscript.

Dual use research of concern

Policy information about dual use research of concern

Hazards

Could the accidental, deliberate or reckless misuse of agents or technologies generated in the work, or the application of information presented in the manuscript, pose a threat to:

No	Yes	
☒	☐	Public health
☒	☐	National security
☒	☐	Crops and/or livestock
☒	☐	Ecosystems
☒	☐	Any other significant area

Experiments of concern

Does the work involve any of these experiments of concern:

No	Yes	
☒	☐	Demonstrate how to render a vaccine ineffective
☒	☐	Confer resistance to therapeutically useful antibiotics or antiviral agents
☒	☐	Enhance the virulence of a pathogen or render a nonpathogen virulent
☒	☐	Increase transmissibility of a pathogen
☒	☐	Alter the host range of a pathogen
☒	☐	Enable evasion of diagnostic/detection modalities
☒	☐	Enable the weaponization of a biological agent or toxin
☒	☐	Any other potentially harmful combination of experiments and agents

ChIP-seq

Data deposition

☒ Confirm that both raw and final processed data have been deposited in a public database such as GEO.

☒ Confirm that you have deposited or provided access to graph files (e.g. BED files) for the called peaks.

Data access links<br>*May remain private before publication.*	http://www.ncbi.nlm.nih.gov/projects/geo/query/acc.cgi?acc=GSE101217
Files in database submission	.bw and .fq files for the following samples:<br>H3K4Me1 ChIP 18h control rep 1<br>H3K4Me1 ChIP 18h control rep 2<br>H3K4Me1 ChIP 18h control rep 3<br>H3K4Me1 ChIP 18h control rep 4<br>H3K4Me1 ChIP 18h Rn7sk KD rep 1<br>H3K4Me1 ChIP 18h Rn7sk KD rep 2<br>H3K4Me1 ChIP 18h Rn7sk KD rep 3<br>H3K4Me1 ChIP 18h Rn7sk KD rep 4<br>H3K27Ac ChIP 18h control rep 1<br>H3K27Ac ChIP 18h control rep 2<br>H3K27Ac ChIP 18h control rep 3<br>H3K27Ac ChIP 18h control rep 4<br>H3K27Ac ChIP 18h Rn7sk rep 1<br>H3K27Ac ChIP 18h Rn7sk rep 2<br>H3K27Ac ChIP 18h Rn7sk rep 3<br>H3K27Ac ChIP 18h Rn7sk rep 4<br>RNA Pol II ChIP 18h control rep 1<br>RNA Pol II ChIP 18h control rep 2<br>RNA Pol II ChIP 18h control rep 3<br>RNA Pol II ChIP 18h control rep 4<br>RNA Pol II ChIP 18h Rn7sk rep 1

RNA Pol II ChIP 18h Rn7sk rep 2
RNA Pol II ChIP 18h Rn7sk rep 3
RNA Pol II ChIP 18h Rn7sk rep 4
Whole cell extract control 18h rep 1
Whole cell extract control 18h rep 2
Whole cell extract control 18h rep 3
Whole cell extract control 18h rep 4
Whole cell extract Rn7sk 18h rep 1
Whole cell extract Rn7sk 18h rep 2
Whole cell extract Rn7sk 18h rep 3
Whole cell extract Rn7sk 18h rep 4

Genome browser session (e.g. UCSC)

No longer applicable.

Methodology

Replicates

ChIP for H3K4me1, H3K27ac and RNA aPol II in control and Rn7sk KD samples.
Whole cell extract in control and Rn7SK KD samples.
Four replicates per condition.

Sequencing depth

The total number of reads was on average 240 million for Pol II samples, 116 million for histone modification samples, and 56 million for input samples. Uniquely mapped reads were on average 9.4 million for Pol II samples, 22 million for histone modification samples, and 42 million for input samples. All ChIP-seq data was single-end 50nt.

Antibodies

Pol II: N-20 from santa cruz biotech (cat N sc-899).
H3K4Me1: Abcam (Ab8895)
H3K27Ac: Abcam (Ab4729)

Peak calling parameters

Histone modification peaks were called using "macs2 callpeak -f BED -g hs -B --call-summits -q 0.01". Whole-cell extract was used as input samples.

Data quality

Between 72,639 and 81,364 H3K27ac peaks were found in the Rn7sk KD replicates.
Between 72,189 and 77,652 H3K27ac peaks were found in the control replicates.
Between 149,838 and 167,062 H3K4me1 peaks were found in the Rn7sk KD replicates.
Between 135,108 and 176,802 H3K4me1 peaks were found in the control replicates.
The replicates showed strong reproducibility. Between 64 and 75% (on average 70%) of the peaks were at least 5-fold enriched over background.

Software

Single-end ChIP-seq reads for PolII, H3K27ac, H3K4me1 ChIP, and WCE (whole cell extract) were quality-trimmed using Trim Galore!, and reads were mapped to the human reference genome (GRCh37/hg19) using bowtie with the parameters "-m 1 –v 2" to generate unique sequence alignments. Potential PCR duplicates were removed with MACS2 'filterdup'.
Genome-wide ChIP-seq read coverage was calculated using 'bedtools genomeCoverageBed', and the genome coverage files in BedGraph format were compressed to bigwig using the UCSC tool 'bedGraphToBigWig'.

Flow Cytometry

Plots

Confirm that:

☐ The axis labels state the marker and fluorochrome used (e.g. CD4-FITC).

☒ The axis scales are clearly visible. Include numbers along axes only for bottom left plot of group (a 'group' is an analysis of identical markers).

☐ All plots are contour plots with outliers or pseudocolor plots.

☒ A numerical value for number of cells or percentage (with statistics) is provided.

Methodology

Sample preparation

Human primary keratinocytes were collected with trypsin, fixed in ice cold 70% ethanol overnight. Next day they were centrifuged, resuspended in PBS with DAPI and analysed

Instrument

LSRFortessa cell analyser (BD bioscience)

Software

FlowJo software (FlowJo LLC)

Cell population abundance

All singlet events were analysed. (no post-sort fractioning was performed in this experiment)

Gating strategy

G1,G0, S and G2M peaks were manually delimited on the 405nm histogram.Fluorescence of each sample was measured at 450/50 405nm. All samples were gated uising forward versus side scatter to eliminate debris.

☒ Tick this box to confirm that a figure exemplifying the gating strategy is provided in the Supplementary Information.
